# Efficacy of recombinant bovine basic fibroblast growth factor gel combined with compound polymyxin B ointment on wound healing after fistulotomy

**DOI:** 10.1097/MD.0000000000046473

**Published:** 2025-12-12

**Authors:** Qing Long, Yan Li

**Affiliations:** a Department of Anorectal Surgery, The Affiliated Hospital, Southwest Medical University, Luzhou, Sichuan Province, China; b Department of Dermatology, The Affiliated Traditional Chinese Medicine Hospital, Southwest Medical University, Luzhou, Sichuan Province, China.

**Keywords:** compound polymyxin B ointment, fistulotomy, recombinant bovine basic fibroblast growth factor gel, skin-sparing debridement, wound healing

## Abstract

This study investigated the effect of recombinant bovine basic fibroblast growth factor (rbFGF) gel combined with compound polymyxin B ointment on wound healing after fistulotomy. This retrospective cohort analysis enrolled 156 patients with simple anal fistulas who underwent fistulotomy between January 2023 and March 2024. The primary outcome was wound healing time. The secondary outcomes included wound pain and exudate scores on postoperative days 3, 7, and 14, recurrence and satisfaction rates, and anal function (Wexner score). Altogether, 156 eligible patients were analyzed, with 71 patients receiving conventional dressing changes (Group 1) and 85 patients receiving rbFGF gel combined with compound polymyxin B ointment (Group 2). The wound healing time was shorter in Group 2 than in Group 1 (*P* < .05). No significant differences were observed in the median wound exudate scores on postoperative days 3 and 7 between the 2 groups, but Group 2 showed lower scores on day 14 (*P* < .05). The pain scores were lower in Group 2 than in Group 1 at all time points (all *P* < .05). At the 12-month follow-up, no recurrence was reported in either group, and the postoperative Wexner scores were not significantly different between the 2 groups, but Group 2 had higher satisfaction rates (94.19% vs 84.51%, *P* < .05). rbFGF gel combined with compound polymyxin B ointment effectively promotes wound healing after fistulotomy, shortens healing time, and reduces postoperative pain.

## 1. Introduction

Anal fistula is an infected tract connecting the anal canal or rectum to the perianal skin,^[1]^ typically presenting with symptoms including perianal pain, swelling, purulent discharge, bleeding, and pruritus.^[2,3]^ As one of the most common diseases in colorectal and anorectal surgeries, anal fistula does not pose a life-threatening risk but considerably impairs the patients’ quality of life, with more severe impacts observed in patients with recurrence, secondary extension, or a sense of urgency.^[4]^

The etiology of an anal fistula is predominantly cryptoglandular infection, where bacteria infiltrate the anal crypts, forming perianorectal abscesses that subsequently rupture or are drained, leaving epithelialized tracts or chronic infectious foci.^[5–7]^ Few cases arise from specific pathologies without overt abscess formation, including Crohn disease, tuberculosis, hidradenitis suppurativa, sexually transmitted infections, or malignancies.^[6,8]^ The incidence of anal fistula is as high as 21 cases per 100,000 population, affecting individuals of all ages but most commonly occurring in patients aged 30 to 40 years, with a male-to-female incidence ratio of 2 to 6 times.^[6]^ Based on the treatment complexity, fistulas are classified as simple or complex. Simple anal fistulas include intersphincteric or low transsphincteric fistulas involving < 30% of the external anal sphincter. Complex anal fistulas encompass high transsphincteric fistulas involving > 30% of the external anal sphincter, as well as suprasphincteric, extrasphincteric, and horseshoe-shaped fistulas, anterior fistulas in women, or those associated with inflammatory bowel disease, radiotherapy, malignant tumors, fecal incontinence, or chronic diarrhea.^[1,9–11]^

For patients with simple anal fistulas and normal anal sphincter function, fistulotomy is recommended.^[7,11]^ Although fistulotomy has a high cure rate, it is often associated with postoperative complications, including incision pain and bleeding, as well as prolonged incision healing time, which delays patients’ return to work. Typical healing times after simple fistulotomy range from 4 to 8 weeks, with significant pain and exudate typically observed in the initial postoperative period.^[12–14]^ A reduction in healing time is considered clinically meaningful in terms of patient comfort and healthcare resource utilization. Effective postoperative wound management not only is related to the improvement of pain relief measures but also can reduce pain and anal fistula recurrence rate as well as shorten hospital stay.^[15]^ Therefore, postoperative wound management of patients with anal fistula is very important. Various techniques for managing postoperative wounds in patients with perianal abscesses or fistulas to reduce postoperative pain and promote wound healing, including nonsteroidal anti-inflammatory drugs, subcutaneous injection of methylene blue at wound edges,^[16]^ premixed nitrous oxide/oxygen mixture,^[17]^ and platelet-rich plasma, have been examined.^[18]^ Nonetheless, randomized controlled trials specifically focusing on topical pharmacologic interventions for post-fistulotomy wounds are still limited, and most existing studies are small-scale or prioritize surgical techniques over adjuvant topical therapies.^[19]^ Moreover, the application of these non-topical methods can be limited by gastrointestinal reactions, complexity, and high costs. In this context, recombinant bovine basic fibroblast growth factor (rbFGF) gel, which has been shown to improve outcomes in recurrent oral ulcers,^[20]^ and compound polymyxin B ointment, which effectively controls infection in chronic refractory wounds,^[21]^ represent promising topical alternatives. Both drugs have demonstrated advantages in terms of promoting the repair of chronic and refractory wounds. Nevertheless, potential risks such as contact dermatitis or allergic reactions to topical antibiotics, as well as concerns regarding antimicrobial stewardship with prolonged use, should be considered.^[22,23]^ Based on these findings, the present study aimed to retrospectively analyze the efficacy of rbFGF gel combined with compound polymyxin B ointment in promoting wound healing after fistulotomy.

## 2. Methods

### 2.1. Participants

The present retrospective study enrolled patients with simple anal fistulas who underwent fistulotomy at the Affiliated Hospital of Southwest Medical University between January 2023 and March 2024. Patients were grouped according to the chronological order of hospital admission, with a total of 156 eligible patients enrolled: 71 received conventional dressing changes (Group 1), and 85 received dressing changes with rbFGF gel combined with compound polymyxin B ointment (Group 2). The inclusion criteria for this study were patients aged 16 to 60 years with simple anal fistulas, regardless of sex, where simple anal fistulas were defined as intersphincteric or low transsphincteric fistulas involving < 30% of the external anal sphincter.^[1,9–11]^ The exclusion criteria were as follows: patients with a history of anal surgery; patients with other anorectal diseases, including severe hemorrhoids (defined as III/IV hemorrhoids or circular mixed hemorrhoids), anal fissures, rectal prolapse, or intestinal tumors; patients with fistulas caused by secondary diseases, including inflammatory bowel disease, tuberculosis, sexually transmitted diseases, or malignant tumors; patients with systemic diseases, including hepatic or renal insufficiency, hypertension, diabetes, long-term use of steroids or immunosuppressive drugs, fecal incontinence, or chronic diarrhea; and pregnant or lactating women. The study was approved by the Ethics Committee of the Affiliated Hospital of Southwest Medical University.

### 2.2. Intervention

The detailed medical history of the patients was documented preoperatively, including symptoms (anal pain, discharge, pruritus, and bleeding) and comorbidities (inflammatory bowel disease, tuberculosis, sexually transmitted infections, malignancies, diabetes, and hypertension), as well as prior anorectal surgical history. All patients underwent digital rectal examination, proctoscopy, and anal sphincter function assessment using the Wexner incontinence score.^[24]^ All patients underwent preoperative magnetic resonance imaging examination of the anal fistula to clarify the internal opening and pipeline shape of the anal fistula and exclude the complex anal fistula. All patients underwent anal fistula resection under spinal anesthesia, and the surgery was performed by senior anorectal surgeons using standard techniques.

Postoperatively, the patients were required to maintain supine positioning for 6 hours before resuming a normal diet, and oral or injectable analgesic drugs were administered according to the postoperative pain severity. Postoperative analgesia followed a stepwise protocol: oral nimesulide (0.1 g, bid, PRN) was used primarily, supplemented with intramuscular tramadol (50 mg, q6h, PRN) if pain persisted. Intravenous ceftazidime (1.0 g, q12h) was administered postoperatively for 48 hours; for patients with penicillin allergy, clindamycin (0.6 g, q12h) was used as an alternative during the same period. Antibiotic use was extended beyond 48 hours only when clinical signs of infection emerged, with adjustments made based on wound culture results. All medication data were extracted from electronic medical records. Bowel movements were restricted for 24 hours, and wound dressing changes were initiated after the patient’s first defecation or within 48 hours postoperatively, with warm water sitz baths (38–42°C) for 15 minutes after defecation. Group 1 received conventional dressing changes. For this group, the patients were placed in the lateral decubitus position to fully expose the surgical wound. The wound and surrounding skin were routinely disinfected with iodophor cotton balls. Then, the necrotic tissue on the wound surface was carefully removed, followed by irrigation with normal saline. Drainage strips were packed into the wound for drainage, which was then covered with a sterile gauze and secured with an adhesive tape. Group 2 received dressing changes with rbFGF gel combined with compound polymyxin B ointment. For this group, after perianal wound disinfection and saline irrigation, rbFGF gel at a dosage of 150 IU/cm^2^ was evenly applied to the wound using cotton swabs, followed by the application of compound polymyxin B ointment with uniform thickness of 1 to 2 mm. Drainage strips were then packed into the surgical wound, which was covered with a sterile gauze and secured with an adhesive tape in the same manner. Both groups underwent daily dressing changes until the anal fistula surgical wound was completely healed.

### 2.3. Outcomes

All assessors responsible for outcome measurement received standardized pre-study training to ensure consistency in scoring. Due to visible differences in dressing residues (gel/ointment vs conventional dressing) and operational steps between the 2 groups, blinding of assessors to group allocation was not implemented. The primary outcome of this study was the wound healing time, defined as complete epithelialization of the wound with firm scarring, absence of an external fistula or perianal discharge, and absence of local discomfort. The secondary outcome measures included wound pain and exudate scores on postoperative days 3, 7, and 14, as well as recurrence and satisfaction rates and anal function. A prespecified set of safety outcomes was also monitored, including the occurrence of contact dermatitis, wound infection, reoperation for bleeding, and other adverse events. Wound pain was assessed using a Visual Analog Scale (VAS), where 0 indicating no pain and 10 indicating the most severe pain. The wound exudate was graded on a severity scale from 0 to 3, with higher scores indicating more severe exudation. At the 12-month follow-up, the recurrence and satisfaction rates and anal function, were evaluated through telephone interviews or outpatient visits. Recurrence was defined as the reappearance of pain, swelling, purulent discharge, tenderness on palpation, or mass at the original fistula site within 12 months after wound healing. Anal function was assessed using the Wexner incontinence score, with 0 indicating a normal function and 20 indicating complete incontinence.^[18]^ Patient satisfaction was evaluated using a 5-point scale (very dissatisfied, somewhat dissatisfied, neutral, somewhat satisfied, very satisfied), with “satisfaction” defined as the sum of “somewhat satisfied” and “very satisfied.”

### 2.4. Statistical analysis

Statistical analyses were performed using SPSS version 25.0 (SPSS Inc., Chicago). Normally distributed continuous variables were expressed as mean ± standard deviation and analyzed using independent samples *t*-tests. Non-normally distributed continuous variables were presented as median with interquartile range and compared using Mann–Whitney *U* tests. Categorical variables were analyzed using Pearson chi-square or Fisher exact test as appropriate. A *P*-value of < .05 was considered statistically significant.

## 3. Results

### 3.1. Patients’ characteristics

**Figure 1.**
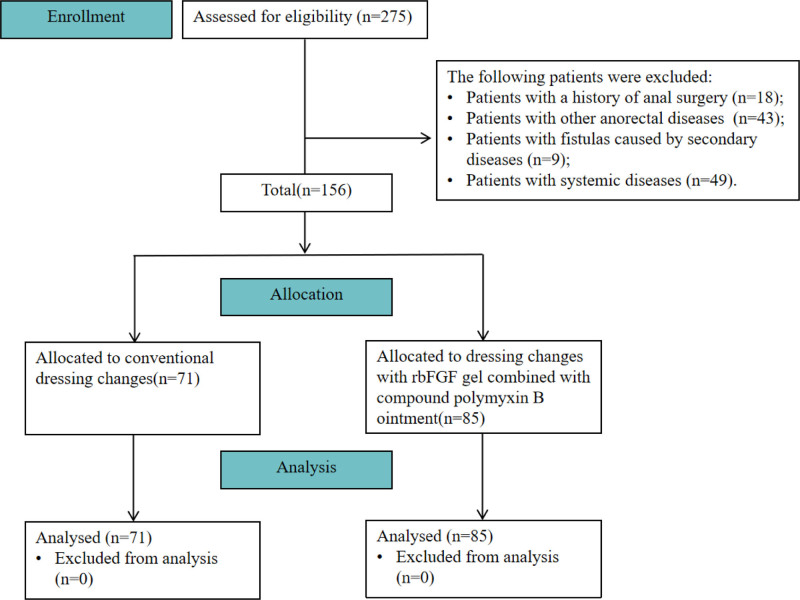
Flowchart of the study design. rbFGF = recombinant bovine basic fibroblast growth factor.

Among the total 156 enrolled patients, 71 received conventional wound dressing (Group 1), whereas 85 were treated with rbFGF gel combined with compound polymyxin B ointment (Group 2). The patient recruitment process is illustrated in Figure 1 using a CONSORT flowchart. No significant differences were observed in sex, age, disease duration, preoperative Wexner incontinence score, or number of incisions (*P* > .05) was observed between the 2 groups (Table 1). None of the patients were lost to follow-up.

**Table 1:** Patients’ characteristics.

Variable	Group 1 (n = 71)	Group 2 (n = 85)	*t*/χ^2^/*Z* value	*P*-value
Age (yr), mean ± SD	42.63 ± 10.14	40.15 ± 10.06	1.527	.129
Sex (male/female)	42/29	51/34	0.011	.915
Disease duration (yr), median (IQR)	3 (1–14)	3 (2–5)	−0.570	.569
Preoperative Wexner incontinence score, median (IQR)	0 (0–0)	0 (0–0)	−0.066	.947
Number of incisions, median (IQR)	1 (1–1)	1 (1–1)	−0.144	.886

IQR = interquartile range, SD = standard deviation.

### 3.2. Primary and secondary outcomes

The wound healing time was shorter in Group 2 than in Group 1 (29.75 ± 2.96 vs 31.45 ± 4.34 days, *P* < .05). No significant differences were observed in the median wound exudate scores on postoperative day 3 between the 2 groups (median [interquartile range]: 2 [2–2] vs 2 [2–2], *P* > .05) and day 7 (1 [1–2] vs 1 [1–1], *P* > .05), but Group 2 showed lower scores than Group 1 on day 14 (0 [0–1] vs 1 [0–1], *P* < .05). Additionally, the pain scores were lower in Group 2 than in Group 1 at all time points (1 day: 2 [2–3] vs 3 [2–3], *P* < .05; 3 days: 1 [1–2] vs 2 [1–3], *P* < .05; and 7 days: 1 [0–1] vs 1 [1–2], *P* < .05). At 12 months postoperatively, the patients were followed up via telephone interviews or outpatient visits. No recurrence was observed in either group. Moreover, no significant difference in the postoperative Wexner scores were observed between the 2 groups (0 [0–0] vs 0 [0–0], *P* > .05), but Group 2 had a higher satisfaction rate than Group 1 (94.19% vs 84.51%, *P* < .05; Table 2). No severe adverse events were recorded in either group. Two patients (2.35%) in Group 2 developed mild contact dermatitis, which resolved after discontinuation of the topical ointment. No wound infections or episodes of bleeding requiring reoperation occurred in either group.

**Table 2:** Outcomes of the patients in Groups 1 and 2.

Variable	Group 1 (n = 71)	Group 2 (n = 85)	*t*/χ^2^/*Z* value	*P*-value
Wound healing time (d), mean ± SD	31.45 ± 4.34	29.75 ± 2.96	2.886	.004
Wound exudate score, median (IQR)				
3 d	2 (2–2)	2 (2–2)	−0.582	.561
7 d	1 (1–2)	1 (1–1)	−1.744	.081
14 d	1 (0–1)	0 (0–1)	−2.243	.025
VAS pain score, median (IQR)				
3 d	3 (2–3)	2 (2–3)	−2.763	.006
7 d	2 (1–3)	1 (1–2)	−3.102	.002
14 d	1 (1–2)	1 (0–1)	−3.145	.002
12-mo postoperative follow-up				
Postoperative Wexner incontinence score, median (IQR)	0 (0–0)	0 (0–0)	−0.114	.909
Recurrence	0	0	–	–
Patient satisfaction	60 (84.51%)	81 (94.19%)	3.981	.046

IQR = interquartile range, SD = standard deviation, VAS = Visual Analog Scale.

## 4. Discussion

The goal of anal fistula surgery is to eliminate the internal opening of the fistula and epithelialized fistula tract, while preserving the function of the anal sphincter,^[7]^ minimizing or avoiding sequelae. However, the treatment remains challenging due to the anatomical complexity of the fistula tract, the intimate relationship with the anal sphincter complex, and the risks of recurrence or incontinence.^[25]^ The surgical approaches for anal fistulas can be classified into sphincter-damaging and sphincter-preserving procedures. Although there are various procedures currently available, no standard procedure is suitable for all cases of anal fistulas; the choice of surgical approach should be determined based on the complexity and depth of the fistula tract, affected muscles, and the surgeon’s experience and preference.^[6,7]^ Fistulotomy is the recommended surgical method for simple anal fistulas,^[7,11]^ as it offers several advantages, high cure and low recurrence rates as well as short operative time. However, for patients undergoing this procedure, the postoperative wound is open, and they still face various issues, such as considerable incision pain, bleeding, excessive discharge, prolonged wound healing time, and risk of anal incontinence. To accelerate wound healing after fistulotomy and prevent the development of postoperative complications, multiple strategies have been developed. Parveen et al^[26]^ used pocket suturing of the wound after fistulotomy for simple anal fistulas, which markedly accelerated the healing time and reduced bleeding, as compared with simple fistulotomy, without increasing postoperative pain or the risk for infection; this finding was also confirmed by Rahman et al.^[27]^ Zhao et al^[28]^ have also showed that one-stage anal fistulotomy surgery combined with shaped skin grafting for low simple intersphincteric anal fistulas can considerably promote wound epithelialization and shorten the healing time. Deng et al^[29]^ used Compound Phellodendron decoction for fumigation and a sitz bath after fistulotomy, which was found to show certain efficacies in promoting wound healing, reducing complications, altering lymphocyte aggregation, and alleviating local inflammatory reactions. Additionally, the topical application of 2% phenytoin, 10% sucralfate, and moist exposed burn ointment has been reported to alleviate post-fistulotomy pain and enhance wound healing.^[19,30,31]^ However, further prospective randomized controlled trials are needed to validate these findings.

Wound healing is a complex and highly coordinated biological process that involves 4 distinct phases – hemostasis, inflammation, proliferation, and remodeling – which collectively promote the restoration of tissue integrity.^[32]^ During this process, the regulation of growth factors and optimization of the local microenvironment are crucial to achieve good healing outcomes. As the most extensively studied member of the fibroblast growth factor family, bFGF exhibits pleiotropic biological activities that promote tissue regeneration through angiogenesis stimulation and mitogenic effects on the epithelial/stromal cells,^[33]^ thereby enhancing regional perfusion and cellular turnover at wound sites. The topical application of rbFGF gel has been shown to be able to repair facial skin lesions in patients with rosacea^[34]^ and to markedly promote healing in patients with recurrent oral ulcers.^[20]^ The compound polymyxin B ointment is a topical antimicrobial preparation composed of polymyxin B sulfate, neomycin sulfate, bacitracin, and lidocaine hydrochloride in a petrolatum base.^[35]^ The triple-antibiotic combination exhibits synergistic antibacterial activity, achieving rapid bactericidal effects while minimizing the risk of resistance through complementary mechanisms.^[21]^ Lidocaine hydrochloride, a local anesthetic, blocks sodium channels in the dermal nerve endings to inhibit nociceptive signal transmission, thereby increasing pain thresholds and alleviating wound discomfort.^[36]^ Multiple studies^[21,37]^ have shown that compound polymyxin B ointment can effectively reduce and control wound infections, accelerate wound repair, and reduce the risk for adverse reactions when used in burns and refractory wounds. A retrospective study by Li et al^[38]^ has demonstrated that compound polymyxin B ointment considerably relieved pain, reduced wound swelling and exudation, and accelerated wound healing in the postoperative care of patients with perianal abscesses.

To promote wound healing after fistulectomy and reduce the inconvenience and cost of wound care, we applied rbFGF gel combined with compound polymyxin B ointment for postoperative wound management.

Our study results showed an association between the combined regimen and shorter wound healing time in Group 2 compared to Group 1 (29.75 ± 2.96 vs 31.45 ± 4.34 days, *P* < .05), suggesting a potential beneficial effect on wound healing duration. Although no established minimal clinically important differences exists for fistula healing time, a reduction of nearly 2 days likely improves patient comfort and resource use. This result may be due to the strong antibacterial activity of polymyxin B, which can effectively reduce bacterial colonization in the wound, prevent the development of wound infection, control the inflammatory response, and accelerate the proliferative phase of wound healing. rbFGF can promote tissue remodeling and wound healing by regulating angiogenesis and cell mitosis, thereby jointly promoting the healing of postoperative wounds of the anal fistula. Additionally, after these 2 drugs cover the wound, they can provide an optimal moist environment for wound healing, allowing keratinocytes to rapidly migrate to the wound surface to complete the reepithelialization process, thereby facilitating a faster healing process.^[39,40]^

Our study results showed no significant difference in the median wound exudate scores between the 2 groups on postoperative days 3 and 7, but Group 2 showed lower scores than Group 1 on day 14 (0 [0–1] vs 1 [0–1], *P* < .05), indicating that the combined dressing change method used in Group 2 could effectively reduce the production of wound secretions, which may be related to the alleviation and control of inflammation by the compound polymyxin B ointment. We also found that the pain scores were lower in Group 2 than in Group 1 at all time points, which may be due to the anti-inflammatory effect of the compound polymyxin B ointment that inhibited the release of inflammatory mediators and the local anesthetic effects of lidocaine present in the compound preparation.

We also found that the pain scores were lower in Group 2 than in Group 1 at all time points. This reduction is likely driven in part by the local anesthetic effect of lidocaine hydrochloride present in the compound polymyxin B ointment, which blocks sodium channels in dermal nerve endings to inhibit nociceptive signal transmission.^[36]^ The anti-inflammatory effect of the polymyxin B formulation, which may suppress the release of inflammatory mediators, probably contributed additionally to the observed analgesic effect. Notably, the reductions in VAS pain scores at all timepoints reach a magnitude considered clinically important according to systematic reviews,^[41]^ suggesting that the intervention offered tangible patient benefits. At 12 months postoperatively, the patients were followed up via telephone interviews or outpatient visits, and no recurrence was observed in either group. Moreover, no significant difference in the postoperative Wexner scores was noted between the 2 groups (0 [0–0] vs 0 [0–0], *P* > .05), but Group 2 had a higher satisfaction rate than Group 1 (94.19% vs 84.51%, *P* < .05). These results indicate that fistulotomy is an effective treatment for simple anal fistulas with high cure and low recurrence rates as well as minimal anal injury, which is consistent with the findings of Cano-Valderrama et al.^[42]^ The association observed with the application of the rbFGF gel combined with compound polymyxin B ointment with reduced wound infections, lower postoperative pain, and accelerated wound healing suggests potential clinical value; however, these findings require confirmation through randomized controlled trials to establish causal efficacy.

The generalizability of our findings is limited to patients with simple anal fistulas and without significant comorbidities. Results may differ in patients with complex fistulas or conditions such as diabetes or inflammatory bowel disease, which can impair wound healing. Future studies should validate these findings in broader patient populations. Furthermore, to balance therapeutic efficacy with antimicrobial stewardship, we suggest an initial period of combined therapy for 1 to 2 weeks, followed by a transition to rbFGF gel monotherapy for continued wound healing if no infection is evident. While a formal cost-effectiveness analysis was beyond the scope of this study, the combination regimen may offer economic benefits by potentially reducing healing time and healthcare visits. Additionally, the simple topical application facilitates easy administration in home-care settings, potentially improving patient compliance and reducing caregiver burden.

The present study had several limitations, including its retrospective single-center design, relatively small sample size, short postoperative follow-up period, and lack of continuous measurement of the anal wound dimensions during follow-up. Further prospective multicenter studies with large sample sizes, extended follow-up periods, and standardized wound measurement methods are needed to improve the research outcomes.

## 5. Conclusion

In patients with simple anal fistulas who underwent fistulotomy, the combined use of rbFGF gel and compound polymyxin B ointment was associated with a significantly shorter healing time (mean difference: 1.70 days; 29.75 ± 2.96 vs 31.45 ± 4.34 days, *P* < .05), lower VAS pain scores at all postoperative time points (1 day: 2 [2–3] vs 3 [2–3], *P* < .05; 3 days: 1 [1–2] vs 2 [1–3], *P* < .05; and 7 days: 1 [0–1] vs 1 [1–2], *P* < .05), and a higher patient satisfaction rate (94.19% vs 84.51%, *P* < .05), demonstrating its clinical value as an effective wound management strategy after fistulotomy. However, as a single-center retrospective non-randomized study with a relatively small sample size (n = 156), these findings require confirmation through large-scale, multicenter randomized controlled trials with extended follow-up to validate long-term efficacy and safety.

## Author contributions

**Conceptualization:** Qing Long, Yan Li.

**Data curation:** Qing Long.

**Investigation:** Yan Li.

**Methodology:** Qing Long, Yan Li.

**Software:** Qing Long.

**Supervision:** Yan Li.

**Writing – original draft:** Qing Long.

**Writing – review & editing:** Yan Li.
